# Supplementary material for: Engagement in moderate-intensity physical activity supports overnight memory retention in older adults
Source: Sci Rep. 2024 Dec 30;14:31873. doi: 10.1038/s41598-024-83336-0 (PMC11686232; doi:10.1038/s41598-024-83336-0)
Supplement: Supplementary file 1 — Supplementary Material 1 [file 41598_2024_83336_MOESM1_ESM.pdf]

Supplemental Material for Engagement in moderate-intensity physical activity supports overnight memory retention in older adults

Miranda G. Chappel-Farley[1,2,3], Destiny E. Berisha[2,3], Abhishek Dave[4], Rachel M. Sanders[5], Chris E. Kline[5], John C. Janecek[2,3], Negin Sattari[6], Kitty K. Lui[7], Ivy Y. Chen[6], Ariel B. Neikrug[6], Ruth M. Benca[3,6,8-9], Michael A. Yassa[2,3,9-10], Bryce A. Mander[2-4,6,9,11]

[1]Department of Psychiatry, University of Pittsburgh, Pittsburgh PA, 15213
[2]Department of Neurobiology and Behavior, University of California Irvine, Irvine CA, 92697, USA
[3]Center for the Neurobiology of Learning and Memory, University of California Irvine, Irvine CA, 92697, USA
[4]Department of Cognitive Sciences, University of California Irvine, Irvine CA, 92697, USA
[5]Department of Health and Human Development, University of Pittsburgh, Pittsburgh PA, 15261
[6]Department of Psychiatry and Human Behavior, University of California Irvine, Irvine CA, 92697, USA
[7]San Diego State University/University of California San Diego, Joint Doctoral Program in Clinical Psychology, San Diego, CA, 92093, USA
[8]Department of Psychiatry and Behavioral Medicine, Wake Forest University, Winston-Salem, NC, 27109, USA
[9]Institute for Memory Impairments and Neurological Disorders, University of California Irvine, Irvine CA, 92697, USA
[10]Department of Neurology, University of California Irvine, Irvine CA, 92697, USA
[11]Department of Pathology and Laboratory Medicine, University of California Irvine, Irvine CA, 92697, USA

Table S1. CHAMPS outcome measures and scoring criteria.

Outcome Variable	Item Number	Coding
Exercise-related Activities		
Frequency of all moderate-intensity exercise-related activities[1]	7, 9, 14-16, 19, 21, 23-26, 29-33, 36- 38, 40	Sum frequency scores/week for each for the subset of activities categorized as moderate intensity (MET >= 3.0)
Duration of all moderate-intensity exercise-related activities[1]	7, 9, 14-16, 19, 21, 23-26, 29-33, 36- 38, 40	Sum the numeric duration variable for the subset of activities categorized as moderate intensity (MET >= 3.0)
Frequency of light-intensity exercise related activities	10, 20, 22, 27, 28, 34, 35, 39	Sum frequency scores/week for each for the subset of activities categorized as light intensity (MET < 3.0)
Duration of all light-intensity exercise-related activities	10, 20, 22, 27, 28, 34, 35, 39	Sum the numeric duration variable for the subset of activities categorized as light intensity (MET < 3.0)
Non-Exertive Activities		
Frequency of non-exertive behavior	1-5, 11-13	Sum frequency scores/week for each activity categorized as non-exertive (allow those with missing data to be included in the sum)
Duration of non-exertive behavior	1-5, 11-13	Sum the numeric duration variable for the subset of activities categorized as non-exertive
Sedentary Behavior		
Frequency of Sedentary Behavior	6, 8, 17, 18	Sum frequency scores/week for each activity categorized as sedentary (allow those with missing data to be included in the sum)
Duration of Sedentary Behavior	6, 8, 17, 18	Sum the numeric duration variable for the subset of activities categorized as sedentary

Table S2. Calculation of outcome variables from CHAMPS for ISM.

Outcome Variable	Item Numbers	Coding
Duration of Sedentary Behavior	6, 8, 17, 18	Sum the numeric duration variable for the subset of activities noted as sedentary. Divide this numeric by 7 to calculate average hours/day. Convert to minutes/day. Divide by 30 to rescale to 30-minute intervals for substitution.
Duration of Non-Exertive Behavior	1, 2, 3, 4, 5, 11, 12, 13	Sum the numeric duration variable for the subset of activities noted as non-exertive. Divide this numeric by 7 to calculate average hours/day. Convert to minutes/day. Divide by 30 to rescale to 30-minute intervals for substitution.
Duration of Light-Intensity Physical Activity	10, 20, 22, 27, 28, 34, 35, 39	Sum the numeric duration variable for the subset of activities noted as light intensity (MET < 3.0). Divide this numeric by 7 to calculate average hours/day. Convert to minutes/day. Divide by 30 to rescale to 30-minute intervals for substitution.
Duration of Moderate-Intensity Physical Activity	7, 9, 14, 15, 16, 19, 21, 23, 24, 25, 26, 29, 30, 31, 32, 33, 36, 37, 38, 40	Sum the numeric duration variable for the subset of activities noted as moderate intensity (MET >= 3.0). Divide this numeric by 7 to calculate average hours/day. Convert to minutes/day. Divide by 30 to rescale to 30-minute intervals for substitution.

Table S3. Categorization of CHAMPS Questionnaire items

Sedentary Behaviors	Non-Exertive Activities	Light-Intensity Activities	Moderate Intensity Activities*
6. Using a computer<br>8. Woodworking/needlework/drawing/arts and crafts<br>17. Play a musical instrument<br>18. Reading	1. Visit friends and family<br>2. Go to the senior center<br>3. Volunteer work<br>4. Attend church<br>5. Attend club or group meetings<br>11. Attend a concert, movie, lecture, or sport event<br>12. Play cards, bingo, or board games with other people<br>13. Shoot pool or billiards	10. Play golf, riding a cart<br>20. Light housework<br>22. Light gardening<br>27. Walk to do errands<br>28. Walk leisurely for exercise or pleasure<br>34. Stretching or flexibility exercises<br>35. Yoga or Tai-chi<br>39. General conditioning exercises, such as light calisthenics or chair exercises	7. Dance<br>9. Play golf, carrying or pulling equipment<br>14. Play singles tennis<br>15. Play doubles tennis<br>16. Ice/roller/in-line skate<br>19. Do heavy housework<br>21. Heavy gardening (spading or raking)<br>23. Work on car, truck, lawn mower, other machinery<br>24. Jog or run<br>25. Walk or hike uphill<br>26. Walk fast or briskly for exercise<br>29. Ride a bike<br>30. Aerobics including rowing or step machines<br>31. Water exercises<br>33. Swim gently<br>32. Swim moderately or fast<br>36. Aerobics or aerobic dancing<br>37. Moderate-to heavy strength training<br>38. Light strength training<br>40. Basketball, soccer, or racquetball

*As published

Table S4. Calculation of CHAMPS variables for ISM with recategorization of items 13 and 17 to light-intensity activity.

Outcome Variable	Item Numbers	Coding
Duration of Sedentary Behavior	6, 8, 18	Sum the numeric duration variable for the subset of activities noted as sedentary. Divide this numeric by 7 to calculate average hours/day. Convert to minutes/day. Divide by 30 to rescale to 30-minute intervals for substitution.
Duration of Non-Exertive Behavior	1, 2, 3, 4, 5, 11, 12	Sum the numeric duration variable for the subset of activities noted as non-exertive. Divide this numeric by 7 to calculate average hours/day. Convert to minutes/day. Divide by 30 to rescale to 30-minute intervals for substitution.
Duration of Light-Intensity Physical Activity	10, 13, 17, 20, 22, 27, 28, 34, 35, 39	Sum the numeric duration variable for the subset of activities noted as light intensity (MET < 3.0). Divide this numeric by 7 to calculate average hours/day. Convert to minutes/day. Divide by 30 to rescale to 30-minute intervals for substitution.
Duration of Moderate-Intensity Physical Activity	7, 9, 14, 15, 16, 19, 21, 23, 24, 25, 26, 29, 30, 31, 32, 33, 36, 37, 38, 40	Sum the numeric duration variable for the subset of activities noted as moderate intensity (MET >= 3.0). Divide this numeric by 7 to calculate average hours/day. Convert to minutes/day. Divide by 30 to rescale to 30-minute intervals for substitution.

Table S5. Pearson's correlation values between average daily minutes spent in each activity type.

	Daily Minutes of Moderate-Intensity Physical Activity	Daily Minutes of Light-Intensity Physical Activity	Daily Minutes of Social Non-Exertive Activity	Daily Minutes of Sedentary Behavior
Daily Minutes of Moderate-Intensity Physical Activity	—	r=0.221, p=0.170	r=0.109, p=0.501	r=0.206, p=0.202
Daily Minutes of Light-Intensity Physical Activity	—	—	r=0.185, p=0.253	r=0.124, p=0.444
Daily Minutes of Social Non-Exertive Activity	—	—	—	r=0.027, p=0.868
Daily Minutes of Sedentary Behavior	—	—	—	—

Table S6. Isotemporal substitution model with items 13 & 17 recategorized as light-intensity: The effect of reallocating 30 min of time spent engaged in sedentary behavior, non-exertive activities, light-intensity physical activity, and moderate-intensity physical activity on overnight negative emotional memory retention (N=40).

Isotemporal Substitution Model	Type of Activity											
	SED			Non-Exertive			LPA			MPA		
	B	SE	p	B	SE	p	B	SE	p	B	SE	p
Model A: Substitution of SED	—	—	—	-0.091	0.058	0.122	-0.089	0.071	0.219	0.019	0.067	0.778
Model B: Substitution of Non-Exertive	0.091	0.057	0.122	—	—	—	0.002	0.053	0.976	**0.110**	**0.048**	**0.029***
Model C: Substitution of LPA	0.089	0.071	0.219	-0.002	0.053	0.976	—	—	—	**0.108**	**0.061**	**0.083^{t}**
Model D: Substitution of MLA	-0.019	0.067	0.778	**-0.110**	**0.048**	**0.029***	**-0.108**	**0.061**	**0.083^{t}**	—	—	—

Data are unadjusted for covariates. Negative values indicate worse overnight negative emotional memory retention. $^{t}p<0.10$, *$p<0.05$, **$p<0.01$
Abbreviations: SED—sedentary behavior; LPA—light-intensity physical activity; MPA—moderate intensity physical activity; B—unstandardized regression coefficient; SE—standard error; p—p-value

Table S7. Isotemporal substitution model with items 13 & 17 recategorized as light-intensity: The effect of reallocating 30 min of time spent engaged in sedentary behavior, non-exertive activities, light-intensity physical activity, and moderate-intensity physical activity on overnight neutral memory retention (N=40).

Isotemporal Substitution Model	Type of Activity											
	SED			Non-Exertive			LPA			MPA		
	B	SE	p	B	SE	p	B	SE	p	B	SE	p
Model A: Substitution of SED	—	—	—	0.030	0.084	0.726	0.032	0.104	0.757	-0.006	0.098	0.956
Model B: Substitution of Non-Exertive	-0.030	0.084	0.726	—	—	—	0.003	0.078	0.971	-0.035	0.071	0.623
Model C: Substitution of LPA	-0.032	0.104	0.757	-0.003	0.078	0.971	—	—	—	-0.038	0.089	0.672
Model D: Substitution of MPA	0.006	0.098	0.958	0.035	0.071	0.623	0.038	0.089	0.672	—	—	—

Data are unadjusted for covariates. Negative values indicate worse overnight neutral memory retention. $^{t}p<0.10$, $^{*}p<0.05$, $^{**}p<0.01$
Abbreviations: SED—sedentary behavior; LPA—light-intensity physical activity; MPA—moderate intensity physical activity; B—unstandardized regression coefficient; SE—standard error; p—p-value

Table S8. Isotemporal substitution model with items 13 & 17 recategorized as light-intensity: The effect of reallocating 30 min of time spent engaged in sedentary behavior, non-exertive activities, light-intensity physical activity, and moderate-intensity physical activity on overnight positive memory retention (N=40).

Isotemporal Substitution Model	Type of Activity											
	SED			Non-Exertive			LPA			MPA		
	B	SE	p	B	SE	p	B	SE	p	B	SE	p
Model A: Substitution of SED	—	—	—	0.085	0.097	0.386	-0.043	0.121	0.726	-0.045	0.114	0.696
Model B: Substitution of Non-Exertive	-0.085	0.097	0.386	—	—	—	-0.128	0.090	0.164	-0.130	0.082	0.122
Model C: Substitution of LPA	0.043	0.121	0.726	0.128	0.090	0.164	—	—	—	-0.002	0.103	0.983
Model D: Substitution of MPA	0.045	0.114	0.696	0.130	0.082	0.122	0.002	0.103	0.983	—	—	—

Data are unadjusted for covariates. Negative values indicate worse overnight positive memory retention. $^{t}p<0.10$, $^{*}p<0.05$, $^{**}p<0.01$
Abbreviations: SED—sedentary behavior; LPA—light-intensity physical activity; MPA—moderate intensity physical activity; B—unstandardized regression coefficient; SE—standard error; p—p-value

Table S9. Pearson's correlation analyses predicting percentage of time spent in each sleep stage, sleep efficiency, and WASO from the frequency of moderate-intensity physical activity.

	TST	NREM1	NREM2	NREM3	REM	SE	WASO
MPA Frequency	r=-0.161, p=0.320	r=0.051, p=0.755	r=0.259, p=0.107	r=-0.158, p=0.329	r=-0.183, p=0.259	r=-0.077, p=0.638	r=0.114, p=0.484

Abbreviations: MPA—Moderate-intensity physical activity; TST— Total sleep time (minutes); NREM—Non-rapid eye movement sleep; REM—Rapid eye movement sleep; SE—Sleep Efficiency; WASO—Wake after sleep onset

Table S10. Pearson's correlation analyses predicting percentage of time spent in each sleep stage, sleep efficiency, and WASO from the duration of moderate-intensity physical activity.

	TST	NREM1	NREM2	NREM3	REM	SE	WASO
MPA Duration	r=-0.250, p=0.119	r=0.128, p=0.432	r=0.257, p=0.109	r=-0.149,p=0.359	r=-0.264,p=0.100	r=-0.119, p=0.466	r=0.198, p=0.222

Abbreviations: MPA—Moderate-intensity physical activity; TST— Total sleep time (minutes); NREM—Non-rapid eye movement sleep; REM—Rapid eye movement sleep; SE—Sleep Efficiency; WASO—Wake after sleep onset
